# Inhibition of succinate dehydrogenase dysregulates histone modification in mammalian cells

**DOI:** 10.1186/1476-4598-8-89

**Published:** 2009-10-22

**Authors:** Ana M Cervera, Jean-Pierre Bayley, Peter Devilee, Kenneth J McCreath

**Affiliations:** 1 Department of Regenerative Cardiology, Centro Nacional de Investigaciones Cardiovasculares Carlos III (CNIC), Madrid, Spain; 2 Department of Human Genetics, Leiden University Medical Center, Leiden, The Netherlands

## Abstract

Remodelling of mitochondrial metabolism is a hallmark of cancer. Mutations in the genes encoding succinate dehydrogenase (SDH), a key Krebs cycle component, are associated with hereditary predisposition to pheochromocytoma and paraganglioma, through mechanisms which are largely unknown. Recently, the jumonji-domain histone demethylases have emerged as a novel family of 2-oxoglutarate-dependent chromatin modifiers with credible functions in tumourigenesis. Using pharmacological and siRNA methodologies we show that increased methylation of histone H3 is a general consequence of SDH loss-of-function in cultured mammalian cells and can be reversed by overexpression of the JMJD3 histone demethylase. ChIP analysis revealed that the core promoter of *IGFBP7*, which encodes a secreted protein upregulated after loss of *SDHB*, showed decreased occupancy by H3K27me3 in the absence of *SDH*. Finally, we provide the first evidence that the chief (type I) cell is the major methylated histone-immunoreactive constituent of paraganglioma. These results support the notion that loss of mitochondrial function alters epigenetic processes and might provide a signature methylation mark for paraganglioma.

## Findings

Forming part of complex II of the respiratory chain, succinate dehydrogenase (SDH) is situated at the intersection of the tricarboxylic acid (Krebs) cycle and oxidative phosphorylation. This combination of functions places SDH at the centre of two essential energy-producing metabolic processes of the cell. Recently, SDH genes have been considered as tumour suppressors since germ line inactivating mutations in the *SDHB, C *and *D *subunit genes can predispose individuals to hereditary paraganglioma (HPGL) [1,2] and phaeochromocytoma [3]. HPGL tumours can be found in the carotid body, a chemoreceptor organ consisting of several cell types [4]. The most predominant cell type in the carotid body is the chief (type I) cell; these cells, of neural crest origin, are arranged in rounded cell nests. The second prominent cell type is the type II glial-like (sustentacular) cell, which surrounds the nest of chief cells. Together, these cells form the striking cell ball of the paraganglion, traditionally referred to as "zellballen" [5].

Although the mechanism(s) linking SDH deficiency to tumour formation remain poorly understood, an activation of the hypoxia pathway is frequently associated with SDH loss of function [6,7]. This results in the stabilization of hypoxia-inducible factor-1α (HIF-1α), a broad-range transcription factor which coordinates cellular adaption to hypoxia [8]. We recently showed that HIF-1α stabilization occurs after chronic silencing of the *SDHB *gene in cultured cells [9], and previous studies have demonstrated that increased cellular succinate, following *SDHD *silencing, inhibits the activity of 2-oxoglutarate-dependent prolyl hydroxylases, master regulators of HIF-1α [10]. Increasing intracellular succinate could, however, also inhibit other 2-oxoglutarate-dependent enzymes, such as the recently identified histone demethylase family of chromatin modifiers [11].

The human genome contains ~30 potential histone demethylases, which are defined by the catalytic jumonji (JmjC) domain [12]. These JmjC histone demethylases (JHDMs) catalyse the 2-oxoglutarate-dependent oxidation of methyl groups in the side chains of the basic amino acids lysine and arginine of histones H3 and H4 [13]. Methylation influences both gene activation and repression, and the effect on chromatin structure depends on the degree of methylation and the specific lysine involved [12]. Histone demethylases are increasingly recognised as playing important roles in many biological processes including development [14], metabolism [15], and cancer [16], and constitute a level of epigenetic control over and above normal transcriptional processes. In this present study we determined whether histone modification was perturbed under conditions of SDH inactivation.

**Figure 1:**
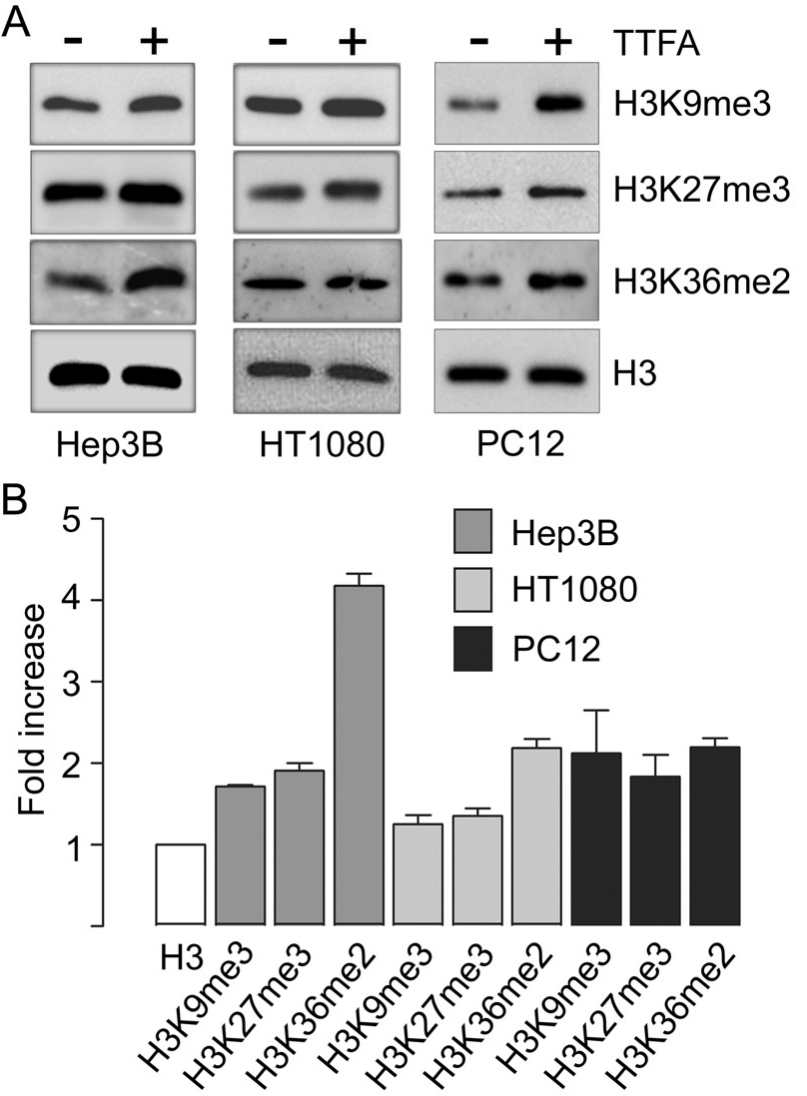
**Pharmacological SDH inhibition increases histone methylation**. (A). Immunoblot analysis of histone methylation in Hep3B, HT1080 and PC12 cells treated for 24 h with 500 μM 2-thenoyltrifluoroacetone (TTFA). In all cases lanes were loaded with 5 μg histone extract, and blots were analysed with the indicated antibodies. Histone H3 total expression was analysed as a loading control. (B) Densitometric analysis of three independent experiments. Fold increase was calculated as the ratio of methylated lysine to H3 control, between treatments.

**Figure 2:**
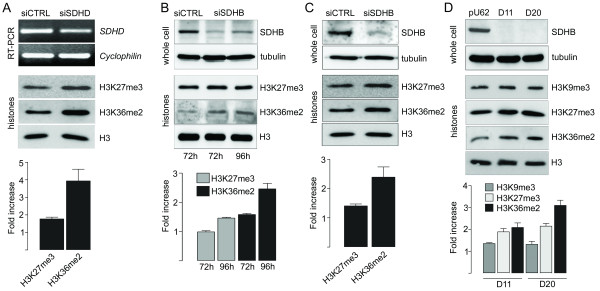
**Succinate dehydrogenase gene inhibition dysregulates histone methylation**. (A) HEK293 cells were transfected in duplicate with ON-TARGET*plus SMART*pool siRNAs targeting *SDHD *or with a non-targeting control (100 nM) for 72 h. One half of the sample was used to prepare RNA and the other half was processed for histone extraction. The silencing efficiency of *SDHD *was analysed by RT-PCR (top panel). Gel lanes were loaded with 5 μg histone extract (bottom panel), and immuno blotting carried out with the antibodies indicated. Histone H3 was used as a loading control. Graph shows densitometric analysis of two independent experiments. (B) Hep3B cells were transfected in duplicate with ON-TARGET*plus SMART*pool siRNAs targeting *SDHB *or with a non-targeting control (100 nM) for 72 and 96 h. One half of the sample was used to prepare whole cell extracts and the other half was processed for histone extraction. Gel lanes were loaded with 20 μg total cell extract (top panel) or 5 μg histone extract (bottom panel), and immuno blotting carried out with the antibodies indicated. Tubulin and histone H3 were analysed as loading controls for whole cell and histone extracts, respectively. Graph shows densitometric analysis of three independent experiments. (C) HEK293 cells were transfected as in (B) and processed for analysis after 96 h. Graph shows densitometric analysis of two independent experiments. (D) Immunoblot analysis of wild-type pU6, and *SDHB*-silenced D11 and D20 cell lines. Samples were processed as described above. Graph shows densitometric analysis of three independent experiments.

**Figure 3:**
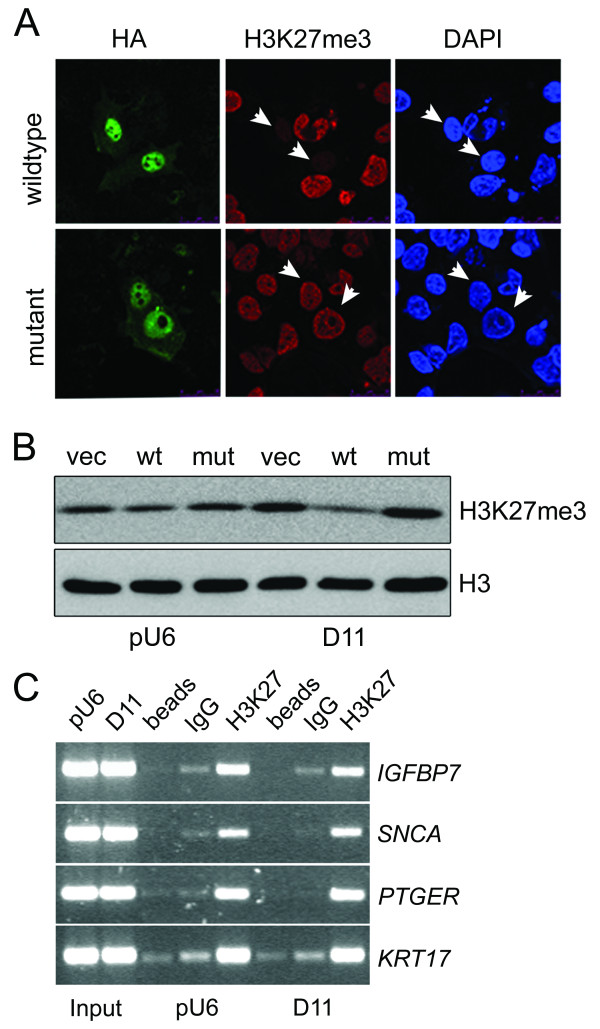
**Functional consequences of increased histone methylation in *SDHB*-silenced cells**. (A) Confocal images of *SDHB*-silenced D11 cells transiently transfected with the overexpression plasmids JMJD3 (wildtype) or JMJD3 mutated (mutant). At 72 h post transfection, cells were fixed and stained with anti-HA and anti-H3K27me3 antibodies; nuclei were stained with DAPI. *Arrows *indicate transfected cells. (B) Immunoblot analysis of cell lines pU6 and D11, transiently transfected for 72 h with pcDNA control (vec), JMJD3 wild-type (wt) or JMJD3 mutated (mut). Results are representative from two independent experiments. **(C) **ChIP analysis of H3K27me3 occupancy of the core promoter regions of *IGFBP7*, *SNCA*, *PTGER*, and *KRT17 *in pU6 and D11 cells. Gels show representative RT-PCR analysis of H3K27me3 and control IgG immunoprecipitates.

Cultured cells were exposed to pharmacological suppression of SDH activity with 2-thenoyltrifluoroacetone (TTFA). Using Western blot analysis with methylation-state-specific antibodies, we determined the steady-state levels of histone 3 methylated on residues K9, K27, and K36. Addition of TTFA resulted in a reproducible increase in global histone 3 methylation in Hep3B and HT1080 human cell lines and also in rat PC12 phaeochromocytoma cells, although the lysine affected and the degree of increase was cell line-dependent (Figure 1A and 1B). We next silenced expression of the endogenous *SDHD *gene in cultured cells. Transient silencing of *SDHD *in HEK293 cells resulted in a significant reduction of *SDHD *mRNA in whole cells (Figure 2A). At the same time, analysis of nuclear histones revealed an increase in steady-state levels of both H3K27me3 and H3K36me2 upon *SDHD *silencing, with H3K36me2 presenting the greatest increase (Figure 2A). To further validate this response we silenced a second SDH gene, *SDHB*. Transient silencing of *SDHB *in Hep3B cells resulted in a robust reduction of SDHB protein as measured by Western blot, and analysis of nuclear histones showed increased steady-state levels of both H3K27me3 and H3K36me2 (Figure 2B). Similar results were obtained after transient silencing of *SDHB *in the HEK293 cell line (Figure 2C), confirming the generality of this response. Moreover, analysis of cells in which *SDHB *was chronically silenced by integrated siRNA (cell lines D11 and D20) [9] revealed a consistent increase in methylated histone residues (Figure 2D). Given that histone methylation is a dynamic phenomenon, we wanted to ensure that the SDH-dependent methylation could be reversed by increasing demethylase activity. We therefore forced overexpression of the H3K27me3-specific Jmjd3 histone demethylase [17] in cells. Transfection of an HA-tagged C-terminal region of Jmjd3, containing the JmjC domain, but not a mutated (non-active) C-terminal region was sufficient to downregulate H3K27me3 levels in Hep3B cells, as shown by double staining with an anti-HA antibody and the methylation-specific anti-H3K27me3 antibody (Figure 3A). Consistently, when overexpressed in the D11 (*SDHB*-deficient) cell line, wild-type but not mutated Jmjd3 downregulated H3K27me3 levels (Figure 3B). Together, these data strongly suggest that SDH gene inactivation leads to a reversible dysregulation of chromatin remodelling by increasing the global level of histone H3 methylation.

We attempted to assess a direct relationship between SDH-induced chromatin alterations and the transcriptional regulation of specific genes. As the full set of genes potentially regulated by this process is unknown, we chose three candidate genes, *SNCA*, *PTGER *and *KRT17*, whose core promoter regions are occupied by H3K27me3, and which have recently been shown to define an epigenetic signature of metastatic prostate cancer [18]. Additionaly, we examined binding at the gene promoter of insulin-like growth factor binding protein 7 (*IGFBP7*), a tumour-related soluble factor whose transcript was highly upregulated in our microarray analysis of *SDHB*-deficient cells [9], and which has been shown to be under epigenetic control [19]. Chromatin immunoprecipitation (ChIP) was carried out with anti-H3K27me3 or IgG control antibody on lysates from control pU6 or *SDHB*-silenced D11 cells. Consistent with previous results [19], subsequent PCR analysis detected H3K27me3 occupancy of the promoters of *SNCA*, *PTGER *and *KRT17*; however, there were no apparent differences between control pU6 and the *SDHB*-deficient D11 cells (Figure 3C). In contrast, H3K27me3 occupancy of the *IGFPB7 *promoter was reduced in D11 compared with pU6 cells (Figure 3C). This was confirmed by quantitative RT-PCR, giving a fold difference in site occupancy of 0.625 ± 0.025 (n = 3). Decreased occupancy would equate to increased transcriptional expression, consistent with our previous results [9], and provides a positive control for future analysis.

**Figure 4:**
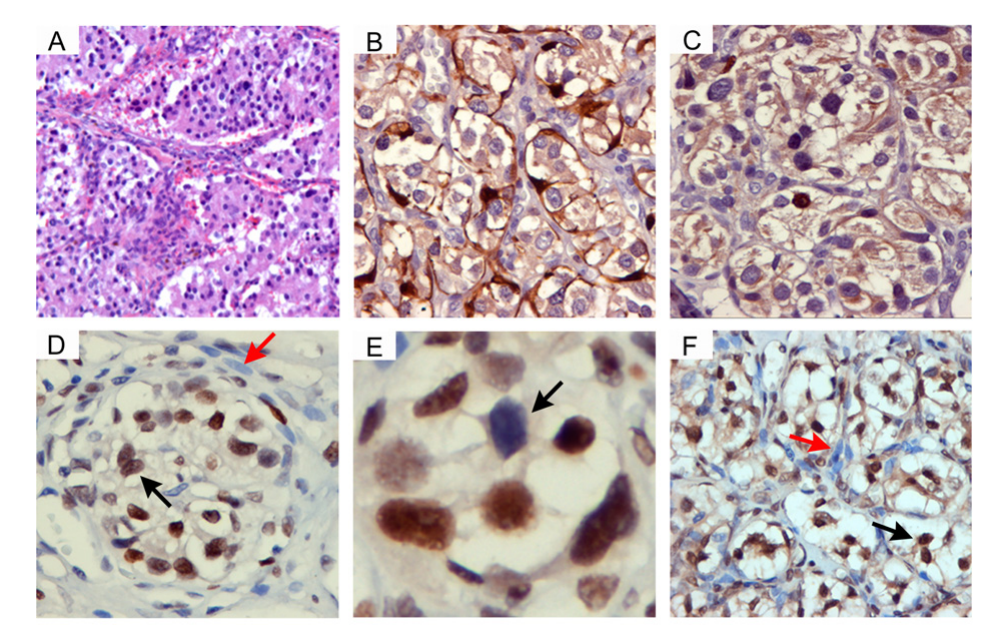
**Immunohistochemical staining for methylated histones in a sporadic carotid body paraganglioma**. (A) Haematoxylin-eosin staining of a sporadic paraganglioma (100× magnification) showing typical cell nest structure. (B) Nuclear and cytoplasmic staining of sustentacular cells using an antibody directed to S100 (400×). Brown staining highlights the typical zellballen structures. (C) Nuclear staining of chief (type I) cells with the tyrosine hydroxylase marker antibody (400×). (D) Anti-H3K27me3 staining (400× with digital enlargement). A clear zellballen structure is visible, showing exclusively nuclear staining of chief cells (dark arrow) but no staining of sustentacular cells (red arrow). (E) High-magnification image of anti-H3K27me3 staining showing heterogeneous nuclear staining of chief cells (arrow). (F) Anti-H3K36me2 staining (400×). There is nuclear and some cytoplasmic staining of chief cells (dark arrow) but no nuclear staining of sustentacular cells (red arrow) and only light (background) staining of the cytoplasm. This specific staining pattern again highlights the zellballen structures. In all cases a sporadic tumour is illustrated, chosen for clarity. SDHD tumours show similar patterns of staining.

Tumours of the carotid body and other paraganglia often retain the general histological pattern of normal paraganglia (Figure 4A). We selected five carotid body paragangliomas and assessed the expression and expected staining pattern of S100, a marker for sustentacular cells and of tyrosine hydroxylase, a marker for chief cells. All tumours tested showed the expected positive (brown) staining pattern (exemplified in a sporadic tumour, Figure 4B and 4C). The tumours were then assessed for histone 3 lysine methylation. As shown in a sporadic paraganglioma tumour, the chief cell fraction showed strong nuclear staining for H3K27me3 (Figure 4D, black arrow). Notably, sustentacular cells (red arrow) showed no nuclear or cytoplasmic staining. A striking feature of H3K27me3 staining in chief cells was its heterogeneity (Figure 4E, black arrow). In contrast, the staining pattern for H3K36me2 was more homogeneous: chief cells showed predominantly nuclear staining (Figure 4F, black arrow) with occasional cytoplasmic staining, while sustentacular cells showed no nuclear staining (Figure 4F, red arrow) and only light, possibly background, staining of the cytoplasm. The staining patterns of both antibodies highlight the "zellball" structure of the tissue. It should be noted that all tumours tested (three sporadic paragangliomas and two *SDHD *tumours) showed similar patterns of staining (not shown). The differential staining of chief cell nuclei would suggest that these cells represent the transcriptionally active component of the paraganglioma. The heterogeneous staining pattern for H3K27me3 in the chief cells is reminiscent of the ultrastructural studies of Grimely and Glenner [20], in which they describe "light" and "dark" chief cells. No specific function has ever been attributed to these two forms, and they may indeed simply be transitory forms of the same cell.

In the present study we have shown that metabolic perturbations within the mitochondrial SDH complex result in a reversible dysregulation of post-translational histone methylation, leading to increased steady-state levels of methylated lysine on histone H3. Product inhibition of the demethylation reaction with succinate is the most likely cause of this dysregulation. The above scenario would predict a non-discriminatory decrease in total cellular demethylase activity following SDH inhibition, orchestrated perhaps by different succinate Ki values for individual demethylases. It is evident that further studies would benefit from genome-wide location analysis (ChIP-on-chip) to survey the underlying chromatin environment associated with SDH dysfunction. As an overture to this analysis, we used ChIP to measure H3K27me3 occupancy at four independent loci, detecting reduced occupancy at the *IGFBP7 *promoter in *SDHB*-deficient cells. Interestingly, recent studies have described the co-existence of H3K4 and H3K27 methylation marks, a so-called bivalent domain, on a subset of developmentally regulated loci in embryonic stem cells [21]. This difference in occupancy between methylated H3K4 and H3K27 could therefore direct increased or decreased transcriptional expression, and provides a plausible explanation for our observations. Of note this study highlights the type I chief cell as the principal immunoreactive cell type for both H3K27me3 and H3K36me2 in the carotid body tumours tested. It would be interesting to see whether all SDH-related tumours show similar staining patterns. Chief cells are the master chemosensory cells of the carotid body and are physiologically complex [22]. Conversely, type II cells lack most of these actions and are generally thought to provide a supporting role to chief cells. Consistent with this notion, multiparameter DNA flow cytometry analysis of *SDHD*-related tumours indicates that chief cells are the neoplastic component of paragangliomas [23]: utilizing S-100 labelling as a selective marker for the sustentacular fraction, this study showed that S-100-labelled cells are diploid, and show retention of the wild-type allele, while loss of the wild-type allele was seen in the S-100-negative fraction. Therefore type II cells can be seen as a non-neoplastic cell population induced as a tumour-specific stromal component of the chief cells.

In summary our initial results demonstrate an epigenetic operation linked to SDH inhibition in mammalian cells, and could provide a paradigm for the investigation of epigenetic processes that may contribute to tumour predisposition in neuroendocrine neoplasia.

## Materials and methods

### Cell culture and transfection

Culture media, fetal bovine serum, and Lipofectamine™ 2000 were from Invitrogen Life Technologies (Carlsbad, CA). All remaining chemicals, unless otherwise stated, were from Sigma Chemical Co. (Poole, UK). Hep3B cells (including cell lines pU6, D11, and D20) were grown in modified Eagle's medium containing 10% FBS, 2 mM L-glutamine, non-essential amino acids, and 1 mM sodium pyruvate. HEK293 cells were maintained in Dulbecco's modified Eagle's medium (DMEM) with 10% FBS and 2 mM sodium pyruvate. Rat phaeochromocytoma PC12 cells were grown in DMEM plus 10% horse serum, 5% FBS and 2 mM L-glutamine. For transient silencing of *SDHB *and *SDHD*, we used Dharmacon ON-TARGET*plus *SMART*pool *siRNA reagents (Thermo Fisher Scientific, Lafayette, CO): catalogue # L-011771-00 targets *SDHB *(NM_003000), catalogue # L-006305-00 targets *SDHD *(NM_003002), and catalogue # D-001810-10-05 is a non-targeting negative control. Cells were transfected with siRNAs (100 nM) using Lipofectamine and were processed for analysis as shown in figure legends. Overexpression plasmids encoding the C-terminal functional domain (aa 1141-1641) of the human *JMJD3 *gene and also a non-functional mutant (His 1388>Ala) were kind gifts from Prof. Gioacchino Natoli (European Institute of Oncology, Milan).

### RNA extraction, chromatin immunoprecipitation and RT-PCR

Total RNA was isolated from cells harvested from t-25 cm^2 ^culture flasks using the RNeasy Mini kit from Qiagen (Valencia, CA). Total cellular RNA (1 μg) was reverse transcribed with 100 Units of Superscript™ II reverse transcriptase (Invitrogen), using oligo-dT primer according to the manufacturer's instructions. Semi-quantitative PCR was then performed using specific oligonucleotide primers for *SDHD *[11] and *cyclophilin *[10]. Chromatin immunoprecipitation was performed using the ChiP kit (Abcam Cambridge, UK), following the protocols provided. Fragmentation of chromatin to >300 bp was verified by electrophoresis. Immunoprecipitated DNA was analysed by PCR using oligonucleotide primers to the promoter regions of the following genes [18]: *PTGER3 *(GGATGGTTGGAGGCTTTGTA and CAGGAAGGTGGCATCAATTT); *SNCA *(GCTGATTGGTGGAAAGGAAA and CACGGTCACAGGTTACAACG) and *KRT17 *(TTGGGGTACAGAAGGGTGAG and TCCCCAGGTTTACACTCCAG). The core promoter region of the *IGFBP7 *gene was analysed using the primers CCCGAGAGGCTTGCTGGAG and AGGCCTGCTGTGGTCTTGGGTGTC, designed using PrimerSelect software (DNAStar, Madison, WI).

### Western blotting, confocal analysis and immunohistochemistry

Preparation of total protein extracts, electrophoresis and membrane transfer were carried out as described [10]. Total histone fractions were prepared using a standard extraction protocol (Abcam). Primary antibodies for immunoblot analysis were purchased as follows: SDHB (Molecular Probes, Invitrogen), β-tubulin (Sigma), H3 and H3K9me3 (Abcam), H3K36me2 and H3K27me3 (Upstate Biotechnology, now Millipore). Protein bands were detected with species-specific peroxidase-conjugated antibodies using the enhanced chemiluminescence method from GE Life Sciences (Piscataway, NJ). For confocal analysis, overexpression constructs were detected using an antibody to the HA peptide (Abcam). Archival formalin-fixed, paraffin embedded paragangliomas (3× sporadic, 1× *SDHD *D92Y, and 1× *SDHD *L139P) were sectioned at 4 μm, and stained with haematoxylin-eosin according to standard protocols, to assess morphology. Further sections were boiled in citrate-buffer pH 6.0 for 10 minutes to retrieve antigens, followed by blocking of endogenous peroxidase activity with hydrogen peroxide, and then used for immunohistochemistry. Sections were incubated overnight (o/n) with an antibody specific for tyrosine hydroxylase (TH) (P40101-0, PelFreez, Arkansas, USA) at 1:500 dilution. After washes, anti-rabbit horseradish peroxidase (HRP) (P0217, Dako, Glostrup, Denmark) secondary antibody was applied for 30 min. The S100 antibody (Z0311, DakoCytomation, Glostrup, Denmark) was used o/n diluted 1:100 in PBS/1% BSA, followed by anti-rabbit HRP (P0217, Dako) for 30 min. An antibody against tri-methylated histone 3 lysine 27 (H3K27me3: ab6002, Abcam) was used o/n diluted 1:50 in PBS/1% BSA, followed by anti-mouse HRP secondary antibody (P0260, Dako) for 30 min. Anti-H3K36me2 antibody (Q16695, Millipore, Amsterdam, Netherlands) was used o/n diluted 1:100 in PBS/1% BSA, followed by anti-rabbit HRP (P0217, Dako) for 30 min. The chromogenic substrate for all secondary antibodies was DAB (K3465, Dako). Sections were further processed by standard techniques.

## Competing interests

The authors declare that they have no competing interests.

## Authors' contributions

AC and KJM conceived and planned the study with help from J-PB and PD. AC performed all cell culture experiments. J-PB carried out immunohistochemistry. PD provided tissue samples. KJM drafted the manuscript with help from AC, J-PB and PD. All authors read and approved the manuscript.
